# The Use of Real-Time Quaking-Induced Conversion for the Diagnosis of Human Prion Diseases

**DOI:** 10.3389/fnagi.2022.874734

**Published:** 2022-04-25

**Authors:** Anna Poleggi, Simone Baiardi, Anna Ladogana, Piero Parchi

**Affiliations:** ^1^Unit of Clinic, Diagnostics and Therapy of the Central Nervous System Diseases, Department of Neuroscience, Istituto Superiore di Sanità, Rome, Italy; ^2^Department of Experimental, Diagnostic and Specialty Medicine (DIMES), University of Bologna, Bologna, Italy; ^3^Programma Neuropatologia delle Malattie Neurodegenerative, IRCCS Istituto delle Scienze Neurologiche di Bologna, Bologna, Italy

**Keywords:** prion disease, Creutzfeldt–Jakob disease, RT-QuIC assay, CSF (cerebrospinal fluid), skin, olfactory mucosa, neurodegenerative diseases, rapidly progressive dementia (RPD)

## Abstract

Prion diseases are rapidly progressive, invariably fatal, transmissible neurodegenerative disorders associated with the accumulation of the amyloidogenic form of the prion protein in the central nervous system (CNS). In humans, prion diseases are highly heterogeneous both clinically and neuropathologically. Prion diseases are challenging to diagnose as many other neurologic disorders share the same symptoms, especially at clinical onset. Definitive diagnosis requires brain autopsy to identify the accumulation of the pathological prion protein, which is the only specific disease biomarker. Although brain post-mortem investigation remains the gold standard for diagnosis, antemortem clinical, instrumental, and laboratory tests showing variable sensitivities and specificity, being surrogate disease biomarkers, have been progressively introduced in clinical practice to reach a diagnosis. More recently, the ultrasensitive Real-Time Quaking-Induced Conversion (RT-QuIC) assay, exploiting, for the first time, the detection of misfolded prion protein through an amplification strategy, has highly improved the “in-vitam” diagnostic process, reaching in cerebrospinal fluid (CSF) and olfactory mucosa (OM) around 96% sensitivity and close to 100% specificity. RT-QuIC also improved the detection of the pathologic prion protein in several peripheral tissues, possibly even before the clinical onset of the disease. The latter aspect is of great interest for the early and even preclinical diagnosis in subjects at genetic risk of developing the disease, who will likely be the main target population in future clinical trials. This review presents an overview of the current knowledge and future perspectives on using RT-QuIC to diagnose human prion diseases.

## Introduction

Prion diseases are rare neurodegenerative disorders of humans and other mammals caused by tissue deposition of an amyloidogenic isoform (PrPSc) of the cellular prion protein (PrPC). In the disease, PrPSc, originating from a stochastic conformational change or acquired from an external source, binds PrPC and mediates its conversion to PrPSc through a seeded conversion mechanism, leading to the accumulation and spreading of the protein aggregates, mainly in the central nervous system (CNS) (Prusiner, 1998). Among neurodegenerative disorders, prion diseases manifest the broadest phenotypic spectrum and the fastest progression leading to death within months or 1–2 years in most cases. Moreover, in contrast to other neurodegenerative disorders, prion diseases include distinctive acquired “infectious” forms caused by ingestion, injection, or transplantation exposure to affected tissues. The high experimental transmission rate of a subgroup of prions led to the demonstration of prion strains, a term underlying the similarities with viral strains. Prion strains were defined as animal or human “isolates” that, after injection into syngeneic hosts, cause diseases with distinct characteristics, such as incubation period, the pattern of PrPSc deposition, and the regional severity of neuropathological changes (Aguzzi et al., 2007; Rossi et al., 2019). A wealth of experimental evidence supports the view that PrPSc enciphers prion strain diversity and that phenotypic heterogeneity in prion disease relates to different structural and physicochemical properties of PrPSc aggregates (Bessen and Marsh, 1994; Telling et al., 1996; Bartz, 2021). These discoveries in prion disease have paved the way to the demonstration that the prion-like transcellular transmission and the causal link between “strains” of protein amyloid aggregates with distinct conformations and phenotypic heterogeneity are common features in neurodegenerative diseases (Jaunmuktane and Brandner, 2020). The broad clinical heterogeneity at onset, often mimicking other rapidly progressive dementias (RPDs), and the variable disease progression and survival times, sometimes overlapping with other more prevalent neurodegenerative dementias, significantly challenge the early clinical diagnosis of prion disease. The long-standing search for a disease-specific biomarker eventually led to the successful development of the prion Real-Time Quaking-Induced Conversion (RT-QuIC), an ultrasensitive assay indirectly revealing minute amounts of PrPSc through an amplification strategy. Here, we will review basic principles, results obtained in human prion diseases, current limitations, and future challenges of the prion RT-QuIC assay.

## Human Prion Disease Phenotypic Spectrum and Classification

### Creutzfeldt–Jakob Disease and Fatal Insomnia

Creutzfeldt–Jakob disease (CJD) is the most common human prion disease. It includes sporadic, genetic, and acquired forms. Sporadic CJD (sCJD), accounting for 85–90% of all cases, is clinically characterized by RPD with ataxia, myoclonus or other neurologic signs and, neuropathologically, by a widespread brain deposition of PrPSc leading to spongiform change, microglial activation, synaptic and neuronal loss, and astrocytic gliosis of variable severity and regional involvement (Zerr and Parchi, 2018). Despite these common features, the disease shows wide phenotypic variability, recognized since its early descriptions. Current sCJD classification comprises six major clinicopathological phenotypes that largely correlate at the molecular level with the genotype (methionine, M or valine, V) at the polymorphic codon 129 of the prion protein gene (*PRNP*) and two PrPSc forms (types 1 and 2) with a distinctive size (21 and 19 kDa) of their proteinase K-resistant core (Parchi et al., 1999a,^2009^, ^2012^). More precisely, each phenotypic variant or “subtype” of sCJD results from a specific codon 129 genotype/PrPSc type combination, with only two exceptions (Table 1). The MM(V)1 subtype includes both MM1 and MV1 cases, given that they are phenotypically indistinguishable. In contrast, the MM2 group comprises two distinctive histotypes with a different topographical distribution of lesions mainly affecting the cerebral cortex (MM2-Cortical or MM2C) or the thalamus (MM2-Thalamic or MM2T). The latter phenotype is also known as the sporadic form of fatal insomnia (Parchi et al., 1999b; Abu-Rumeileh et al., 2018). It is also noteworthy that about 35% of sCJD cases show the co-occurrence of PrPSc types 1 and 2, mainly affecting the subgroup of sCJD subjects carrying MM at codon 129 (Parchi et al., 2009). In these “mixed” subtypes, pathological features of two “pure” subtypes co-occur, with the predominant lesions reflecting the most prevalent PrPSc type in the brain (Parchi et al., 2009). Genetic CJD (gCJD), also including the familial form of fatal familial insomnia (FFI), a prion disease subtype with distinctive clinicopathological features, is the second most common human prion disease, accounting for 10–15% of CJD cases. Patients with gCJD carry point mutations or octapeptide repeat insertions (OPRI) in *PRNP* (Capellari et al., 2011). The clinicopathological variability of gCJD essentially reproduces that of sCJD since the disease phenotype is also primarily determined by the PrPSc types 1 and 2 in conjunction with the codon 129 genotype in the mutated allele (Baiardi et al., 2021). Nevertheless, there are a few significant exceptions to this general rule, given that a few mutations co-distribute with a phenotype that is divergent from that expected from the *PRNP* haplotype (i.e., genotype determined by the 129 polymorphism and the mutation) and PrPSc type combination (Baiardi et al., 2021). Iatrogenic CJD (iCJD) resulted from the human-to-human transmission of CJD through medical procedures (Brown et al., 2012). The principal sources of infectious prions have been contaminated dura mater graft, and human-derived pituitary growth hormone extracted from tissues obtained post-mortem. Others included contaminated neurosurgical instruments, human derive-gonadotropins, corneal grafts, and blood transfusions from patients with variant CJD (vCJD). Although, as in sCJD, the pathological phenotype of iCJD largely depends on the *PRNP* codon 129 genotype and the type of PrPSc accumulating in the brain, the phenotypic spectrum of iCJD, likewise gCJD, does not fully reproduce that of the sporadic form. Of note, while, to date, there have been no proven iCJD cases showing the MM2C, MM2T, or VV1 histotype, a “plaque-type” histotype linked to MM at codon 129 has been consistently observed in iCJD but not in sCJD (Shimizu et al., 1999; Hoshi et al., 2000; Kretzschmar et al., 2003; Yamada et al., 2009; Kobayashi et al., 2014).

**TABLE 1:** Phenotypic spectrum of human prion disease.

Group	Etiology	Frequency	Phenotype(s)	Relevant annotations
CJD/FI	Sporadic	80–85% of all cases	sCJD MM(V)1	Typical/myoclonic variant (most frequent)
			sCJD VV2	Ataxic/cerebellar variant
			sCJD MV2K	Cerebellar kuru-plaque variant
		Rare	sCJD MM2C	Cortical variant
			sCJD MM2T	Thalamic variant (also known as sporadic fatal insomnia)
			sCJD VV1	Cortico-striatal variant
	Genetic	10–15% of all cases	gCJD	Various phenotypes according to PrP*^Sc^* type and *PRNP* haplotype overlapping with the sCJD spectrum
			FFI	Thalamic variant, linked to the D178N-129M haplotype
		Rare	Atypical	A minority of phenotypes that diverge from what expected from the PrP*^Sc^* type/*PRNP* haplotype combination
	Acquired		np-iCJD, 129MM	Similar to sCJD MM(V)1 but with amyloid plaques in subcortical white matter
			p-iCJD, 129MM	Widespread kuru plaques in the brain
			iCJD MV2K	Virtually indistinguishable from sCJD MV2K
			vCJD	Florid amyloid plaques. significant involvement of lympho-reticular tissues beside CNS; almost disappeared
VPSPr	Sporadic		VPSPr	Low resistance of PrPSc aggregates to PK-digestion. especially in 129VV carriers (most frequent genotype)
Inherited amyloidosis	Genetic		GSS	CNS PrP-amyloid plaques; secondary tauopathy
			PrP-CAA	Linked to stop-codon truncating *PRNP* mutations
			PrP-SA	Linked to stop-codon truncating *PRNP* mutations; systemic form of PrP amyloidosis

*CJD, Creutzfeldt–Jakob disease; sCJD, sporadic CJD; gCJD, genetic CJD; iCJD, iatrogenic CJD; np-iCJD, non-plaque-type iCJD; p-iCJD, plaque-type iCJD; vCJD, variant CJD; FI, fatal insomnia; FFI, fatal familial insomnia; VPSPr, variably protease-sensitive prionopathy; GSS, Gerstmann–Sträussler–Scheinker disease; CAA, cerebral amyloid angiopathy; SA, systemic amyloidosis; CNS, central nervous system.*

Variant CJD is an acquired, zoonotic prion disease caused by oral exposure to food contaminated with material from bovine spongiform encephalopathy (BSE)-affected cattle. Since 1995, when the first identification of the disease in the United Kingdom occurred (Will et al., 1996), there have been approximately 230 individuals affected by vCJD, mainly in the United Kingdom (Will and Ironside, 2017). However, the disease has become exceedingly rare in recent years, following the decline of BSE in cattle. Pathological hallmarks of vCJD include the presence of widespread and numerous florid plaques, characterized by a central eosinophilic amyloid core and a pale periphery of radiant fibrils surrounded by a corona of vacuolation, and prion protein (PrP) accumulation as small cluster plaques (Ritchie and Ironside, 2017). Given the “peripheral” pathogenesis and the lymphotropism of the BSE strain, PrPSc is also present in relatively high amounts (i.e., in comparison to other CJD phenotypes) outside the CNS, especially in the lymphoreticular tissues (Wadsworth et al., 2001).

### Variably Protease-Sensitive Prionopathy

Variably protease-sensitive prionopathy (VPSPr) is the most recently recognized sporadic prion disease and owes its name to the peculiar properties of its PrPSc aggregates. Although VPSPr may affect subjects harboring each of the three genotypes at codon 129, it is most frequently linked to valine homozygosity (129VV) (Head et al., 2009, 2013; Zou et al., 2010; Assar et al., 2015). The clinical phenotype is often consistent with an unspecific, Alzheimer-like, or frontotemporal-like dementia. However, two patients with autopsy-confirmed VPSPr and a clinical diagnosis of amyotrophic lateral sclerosis have also been reported (Vicente-Pascual et al., 2018). Spongiform change is characterized by non-confluent vacuoles of intermediate size [i.e., larger than in typical sCJD MM(V)1, but smaller than in sCJD MM2C]. The lesion profile shows a predominant involvement of cerebral cortices, striatum, and thalamus, while the cerebellum is variably affected and the hippocampus relatively spared. PrP immunostaining reveals micro plaques in the molecular layer of the cerebellum surrounded by clusters of granular deposits (i.e., target-like pattern). Western blot analysis of PrPSc shows a ladder-like profile of at least 5 proteinase K-resistant fragments migrating in the 7–26 kDa range (Zou et al., 2010). The lack or the minimal representation of the diglycosylated PrPSc isoform is another distinctive feature of VPSPr. Furthermore, both the amount and the resistance of PrPSc to proteinase K digestion are lower in VPSPr than in sCJD (Saverioni et al., 2013). Proteinase K-resistance is also significantly influenced by the codon 129 genotype: it is lowest in VPSPr 129VV, highest in 129MM, and intermediate in 129MV (Zou et al., 2010; Baiardi et al., 2022).

### Inherited Prion Protein-Amyloidosis

Inherited PrP-amyloidosis encompasses a distinct group of genetic prion diseases characterized by the accumulation of an internal PrPSc fragment, truncated at both C- and N-termini, in the form of amyloid plaques or cerebral amyloid angiopathy (Ghetti et al., 2018). Depending on the causative mutation, the molecular mass of the abnormal PrPSc fragment may range from 6 to 11 kDa and is occasionally associated with other PrP truncated species with higher molecular mass. The broad phenotypic heterogeneity in this group, observed even within family members, depends on the several disease-associated genetic mutations, including point, stop-codon *PRNP* mutations, 7–12 OPRI, and duplication in the hydrophobic domain, the specific *PRNP* haplotype, and other yet undefined individual factors. Gerstmann–Sträussler–Scheinker (GSS) is the most prevalent inherited PrP-amyloidosis and the first genetic prion disease linked to a *PRNP* mutation (Hsiao et al., 1989). Clinically, GSS often manifests slowly progressive ataxia, pyramidal signs, and late dementia. The accumulation of amyloid PrP plaques in the CNS, especially in the cerebral and cerebellar cortices, represents its pathological hallmark (Ghetti et al., 2018). Spongiform change can be either absent (more often), limited to small foci or widespread. Finally, it is noteworthy that the GSS phenotype also comprises tau pathology in the form of neurofibrillary tangles and dystrophic neurites around the PrP plaques (Ghetti et al., 2018). The more recent discovery of premature truncating mutations in *PRNP* linked to familial prion diseases has significantly expanded the phenotypic spectrum of PrP-amyloidosis (Table 1). Besides GSS, *PRNP* truncating mutations have been linked to cerebral amyloid angiopathy (Ghetti et al., 2018) and, more recently, even found in patients with chronic diarrhea, progressive autonomic failure, and peripheral polyneuropathy in association with widespread abnormal PrP deposition in both peripheral organs and CNS (Matsuzono et al., 2013; Mead et al., 2013; Capellari et al., 2018).

### Challenges to the Early Clinical Diagnosis

The broad clinical heterogeneity at onset, often mimicking other RPDs, and the variable disease progression and survival times, sometimes overlapping with other more prevalent neurodegenerative dementias (e.g., Alzheimer’s disease), significantly challenge the early diagnosis and clinical management of patients with prion disease (Baiardi et al., 2018). Yet, a reliable early diagnostic formulation is crucial to rule out other rapidly progressive and potentially treatable neurological syndromes, reduce the risk of iatrogenic transmission, and improve epidemiological disease surveillance. Besides the search of mutations in *PRNP*, which represent the main diagnostic tool for familial prion disease, electroencephalographic findings, brain-derived cerebrospinal fluid (CSF) protein assays serving as surrogate markers for neuronal damage, and diffusion-weighted magnetic resonance imaging (DW-MRI), have provided the primary support for the clinical diagnosis of prion disease in the past 20 years. However, these biomarkers provide a suboptimal diagnostic accuracy and show a significant variability across diseases and their subtypes. Accordingly, the in vitam diagnosis of sCJD has become ‘‘probabilistic’’ (‘‘possible’’ or ‘‘probable’’), based on the evaluation of neurological symptoms and signs and the results of the above-mentioned ancillary tests^1^ (Table 2). Periodic sharp-wave complexes (PSWCs) represent the most characteristic electroencephalographic anomaly detected in sCJD (Wieser et al., 2006). However, it is only found in about 65% of patients, resulting in a low diagnostic sensitivity (Steinhoff et al., 2004). Moreover, PSWCs may also be recorded in other RPDs (e.g., various encephalitis, hepatic encephalopathy, and lithium intoxication) (Karnaze and Bickford, 1984; Smith and Kocen, 1988; Steinhoff et al., 1996, 2004; Tschampa et al., 2001), resulting in a suboptimal specificity. As an additional limitation, PSWCs typically only show up during the clinical course of the disease, 2–3 months after onset (Hansen et al., 1998; Wieser et al., 2004), and may even disappear in the pre-terminal phase (Wieser et al., 2004). Hence, an electroencephalographic recording done too early or, less commonly, too late in the course of the disease may miss the PSWCs. Finally, PSWCs are commonly recorded only in the “typical” form of the disease [i.e., the MM(V)1 subtype], not in the other sCJD subtypes (Parchi et al., 1999a). Brain MRI has played a pivotal role in the clinical diagnosis of sCJD since the introduction of the Fluid Attenuated Inversion Recovery and DWI techniques. The step forward has significantly increased the MRI’s diagnostic accuracy reaching a 91–96% sensitivity and a 88–95% specificity (Shiga et al., 2004; Young et al., 2005; Vitali et al., 2011; Carswell et al., 2012). The DWI sequence has a higher sensitivity than Fluid Attenuated Inversion Recovery, particularly for the early detection of signal hyperintensity and its evaluation at the cortical level (Shiga et al., 2004; Tschampa et al., 2007; Vitali et al., 2011). The increased signal in DWI scans correlates with the underlying presence and degree of spongiform change (Manners et al., 2009). In general, typical findings in sCJD patients are signal hyperintensity in the deep gray matter (caudate nucleus and putamen, less commonly the thalamus), the cerebral cortex, or both (Zerr et al., 2009). As the disease progresses, the signal intensity usually increases and extends to additional brain regions (Ukisu et al., 2005; Eisenmenger et al., 2016). These signal abnormalities reflect the heterogeneity of the lesion profiles among sCJD subtypes in terms of spatial pattern and its evolution during disease progression (Baiardi et al., 2019a). The typical abnormal sCJD signal profile comprises an increased signal in Fluid Attenuated Inversion Recovery or DWI sequences in at least two cortical regions (temporal, parietal, or occipital) or at the level of caudate and putamen or in both areas. This profile has optimal diagnostic accuracy in the differential diagnosis with other RPDs and is part of the current diagnostic criteria for sCJD (Hermann et al., 2021). DW-MRI may disclose a typical, almost subtype-specific, lesion pattern in a subset of cases, including a high signal intensity in the basal ganglia and thalamus in the VV2 subtype, a prominent, often isolated, cortical signal hyperintensity in subjects MM2C or VV1, and a marked asymmetry of the signal hyperintensity in the MM(V)1 variant (Meissner et al., 2009; Bizzi et al., 2021). On the other hand, DW-MRI may be unrevealing in 5–10% of cases, depending on the disease subtype and the time from disease onset, and yields a variable percentage of false-positive scans in RPDs with non-prion etiologies (Zerr et al., 2009). In this regard, it is worth noting that CJD surveillance centers, likely as a consequence of improved technology and increased awareness of DWI hyperintensities, have been witnessing an increase in referrals due to MRI findings of isolated cortical ribboning in patients with seizures, hyponatremia, Wernicke’s encephalopathy or even ischemia, often in the absence of typical clinical features of CJD. The analysis of CSF offers essential support to the clinical diagnosis of sCJD and the exclusion of an inflammatory pathology of the CNS. Routine cytological and biochemical examinations of CSF from affected patients are typically free of significant abnormalities (Olsson, 1980). One-third of cases show a slight non-specific increase in total protein concentration, and, occasionally, a mild pleocytosis or oligoclonal bands are also present (Jacobi et al., 2005). Among the CSF markers of CJD, proteins 14-3-3 and total tau represent validated surrogate markers of neuronal damage (Hermann et al., 2021). The 14-3-3 protein assay exhibits a diagnostic sensitivity of 85–95% and a specificity of 74–96% (Zerr and Parchi, 2018). The finding of elevated concentrations of 14-3-3 in an appropriate clinical setting supports the diagnosis of CJD. However, given that this cytosolic protein is released into the CSF following non-specific neuronal damage (Satoh et al., 1999), other pathologies, including other neurodegenerative RPDs, metabolic encephalopathies, stroke, paraneoplastic syndromes, tumors, may also yield a positive test (Saiz et al., 1999; Chapman et al., 2000; Burkhard et al., 2001; Lattanzio et al., 2017). Patients with CJD usually show an increase in levels of 14-3-3 as the disease progresses, unlike those with acute neuronal damage, where the trend is the opposite (Sanchez-Juan et al., 2007). Of note, the assay demonstrated a lower sensitivity and higher incidence of false negatives for sCJD subtypes characterized by slower progression and longer disease duration (such as MM2 and MV2K) (Castellani et al., 2004; Sanchez-Juan et al., 2006; Lattanzio et al., 2017). High values of total tau (>1,200–1,300 pg/ml) have shown a slightly higher diagnostic accuracy for all subtypes of sCJD. However, likewise 14-3-3, the dosage of total tau is not exempt from false positives and negative readouts (Zerr and Parchi, 2018). Indeed, patients with Alzheimer’s disease and with inflammatory or neoplastic disorders of the CNS may also show high concentrations of total tau in CSF (Constantinescu et al., 2016; Olsson et al., 2016; Lehmann et al., 2019). Finally, similarly to 14-3-3, a reduced sensitivity has been demonstrated in the MM2 and MV2K subtypes, VPSPr, and early disease stages (Cohen et al., 2016; Lattanzio et al., 2017).

**TABLE 2:** Current diagnostic criteria for surveillance of sCJD (updated January 01, 2017).

	Clinical criterion	Supportive laboratory investigation(s)
Definite	Progressive neurological syndrome	Neuropathological confirmation, OR Immunohistochemical confirmation, OR Biochemical confirmation
Probable	Rapidly progressive cognitive impairment AND at least two of the following: (1) Myoclonus. (2) Visual or cerebellar problems. (3) Pyramidal or extrapyramidal features. (4) Akinetic mutism.	Generalized periodic complexes at EEG, OR Positive CSF 14-3-3 protein, OR High signal in caudate/putamen on MRI brain scan or at least two cortical regions (temporal, parietal, and occipital) either on DWI or FLAIR
	Progressive neurological syndrome.	Positive RT-QuIC assay in CSF or other tissues
Possible	Rapidly progressive cognitive impairment AND at least two of the following: (1) Myoclonus. (2) Visual or cerebellar problems. (3) Pyramidal or extrapyramidal features. (4) Akinetic mutism AND disease duration <2 years	None

*EEG, electroencephalography; CSF, cerebrospinal fluid; MRI, magnetic resonance imaging; DWI, diffusion-weighted imaging; FLAIR, fluid attenuated inversion recovery; RT-QuIC, real-time quaking-induced conversion.*

## The Real Time Quaking-Induced Conversion Assay

### Basic Principles

RT-QuIC is the first antemortem technique that can reliably detect misfolded PrP, the only available disease-specific biomarker for prion disease. The assay demonstrated high specificity and variable sensitivity for human prions, depending on the disease subtype and the applied protocol. RT-QuIC exploits the self-replicating (seeding) ability of pathogenic PrPSc as an amplification strategy to ultra-sensitively detect the limited amounts of the abnormal protein present in CSF and other tissues.

The development of RT-QuIC and other similar seeding assays (e.g., protein misfolding cyclic amplification) stem from the protein-only hypothesis of prion replication (Prusiner, 1998) and the demonstration that aggregated species of PrP can be generated in a cell-free assay (Kocisko et al., 1994). This seminal result was obtained by incubating an excess of partially denatured PrPSc, isolated from prion-infected hamsters, with 35S-labeled recombinant PrPC (rPrP) derived from uninfected hamster brains. The subsequent demonstration of proteinase K-resistant labeled PrP proved the *de novo* formation of misfolded species (Kocisko et al., 1994). Using cyclic sonication and bacterially expressed rPrP in vast excess to facilitate the conversion into its pathogenic counterpart and an automated system to guarantee reproducibility, Atarashi et al. (2007) developed the first QuIC assay. In this reaction, minute amounts of seed derived from brain homogenates were sufficient to trigger a significant aggregation of the excess of the rPrP in a reasonable time. By combining the QuIC cyclic amplification with the real-time detection of the products by thioflavin T (ThT), Wilham et al. (2010) later developed the currently used RT-QuIC set-up.

**FIGURE 1:**
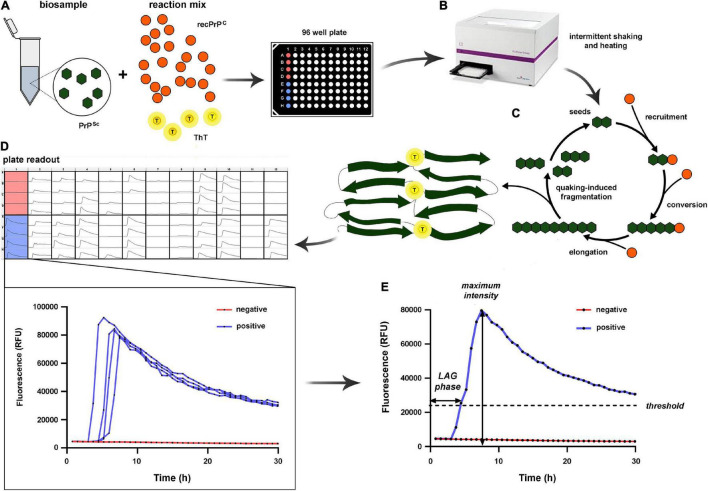
Schematic illustration of the RT-QuIC assay. **(A)** The reaction mix includes the biosample to evaluate for the presence of PrPSc seeds, a relatively large amount of recombinant PrPC (recPrP*^C^*), and thioflavin-T (ThT). **(B)** Each sample is loaded in quadruplicate (e.g., column 1, rows A–D are loaded with the same sample) in a 96-well plate; then the plate is incubated at a given temperature, and samples undergo intermittent rest/shaking cycles. **(C)** Overview of the reaction process: PrPSc seeds progressively elongate through recruitment and conversion of rPrP. The novel PrPSc oligomers are partially fragmented by quaking, producing novel seeds while, in part, they continue to elongate up to the status of protofibrils and eventually fibrils. By acquiring amyloid proprieties, the protofibrils/fibrils bind ThT, which produces a fluorescent signal monitored in real-time. **(D)** Plate readout at the end of the reaction. **(E)** On the left, quantitative evaluation of two exemplificative cases from column 1: lack of signal modifications over 30 h in A–D rows (negative sample, highlighted in red), and increase of fluorescent signal in E–H rows (positive sample, highlighted in blue). Each dot on the curves corresponds to a fluorescent signal reading. On the right, the positive and negative replicates are merged to show the two most significant kinetic parameters of the reaction, namely the LAG phase (time to reach the threshold) and the maximum intensity (the peak of the fluorescent signal).

In the prion RT-QuIC assay, a patient’s biological sample containing PrPSc (seed) is mixed in 96-well plates with an excess of rPrP as a substrate, a standard buffer, and ThT, a fluorescence emitting dye with a strong affinity with protein amyloid structures (Wilham et al., 2010; Atarashi et al., 2011; Orrú et al., 2015a) (Figure 1). The plate is then incubated at various temperatures (from 42 to 55°C) depending on the specific protocol and subjected to cycles of double orbital shaking and rest inside a fluorescence microplate reader. While incubating, the specimen PrPSc seed induces a conformational change of the rPrP substrate, resulting in aggregation and amyloid fibril formation (Atarashi et al., 2011). The ThT fluorescence emission, recorded in real-time during the entire incubation period, allows the detection of these aggregates when formed. A typical sigmoidal curve of fluorescence indicates the positivity of the test. The amyloid formation follows an exponential increase after an initial lag phase of variable duration with only a background signal. Shaking, another critical factor for the reaction, promotes the fragmentation of aggregates and the formation of novel, free reactive seeds, accelerating the amyloid fibril formation. A decline in fluorescence is observed at the end of the assay, likely due to ThT binding to amyloid fibrils which leads to fluorescence self-quenching and fibril compaction (Lindberg et al., 2017) (Figure 1).

There is a significant influence of the disease subtype/prion strain on the outcome of the RT-QuIC, determining a variable diagnostic performance of the assay in human prion disease (see the section on disease diagnostics). Besides, pre-analytical factors, the recombinant substrate, and the tissue matrix may also affect the assay’s results.

### The Effect of Pre-analytical Factors

The concentration of particular cellular or molecular components in the tested sample matrices affects the prion RT-QuIC performance. For example, an increased total protein concentration in CSF > 1.0 g/L or a white cell count >10 × 10^6/L, can lead to false-positive results (Green, 2019). In contrast, red cells CSF contamination has an opposite effect and may result in a potentially false negative outcome (Cramm et al., 2016; Foutz et al., 2017). An excessive volume of human CSF can also inhibit the assay (Xiao et al., 2018), probably due to the presence of unknown factors that hamper amyloid formation in the RT-QuIC reaction. A high concentration of brain homogenate has a similar effect (Wilham et al., 2010; Mori et al., 2016), probably due to the inhibition exerted by endogenous brain polar lipids (Hoover et al., 2017).

In contrast, other classic pre-analytical variables known to potentially affect biomarker performance in biofluids, such as sample storing at room temperature or 4°C for up to 8 days or repeated freeze-thaw cycles, seem to have minimal or no effects on the RT-QuIC outcome (Cramm et al., 2016). However, these findings should not be considered conclusive and await replication in confirmatory studies.

### The Effect of Substrate and Other Analytical Factors

Recombinant prion proteins, expressed in *Escherichia coli*, represent the substrates of choice for RT-QuIC. The development and production of suitable rPrP substrates, rapidly converting to amyloid in the presence of prion seeds but not prone to self-aggregation, is mandatory for optimal performance. Early RT-QuIC assays employed the full-length Syrian hamster rPrP (Ha rPrP23-231) or the full-length human rPrP (Hu rPrP23-231) (Wilham et al., 2010; Atarashi et al., 2011) as substrates. These proteins were stable and performed well, although not optimally when seeded with brain or CSF from typical sCJD (McGuire et al., 2012) but showed much lower sensitivity with samples from vCJD (Peden et al., 2012) or other rare and atypical sCJD subtypes (Lattanzio et al., 2017). Therefore, there have been attempts to improve the sensitivity for these specimens using modified protocols (i.e., sodium dodecyl sulfate concentration in the reaction buffer, temperature of incubation, and shaking speed) (Orrú et al., 2016), and more reactive substrates such as truncated hamster rPrP (Ha rPrP90-231) (Orrú et al., 2015a) in the so-called improved QuIC (IQ-CSF). The use of the N-terminal truncated hamster rPrP as substrate associated with modified parameters, including the incubation temperature of 55°C, the shaking speed of 700 rpm, and the sodium dodecyl sulfate concentration of 0.002%, produced a shorter experimental time with an increase in sensitivity of the test for sCJD in comparison to the first-generation RT-QuIC assay. The reason why the Ha rPrP90-231 substrate produces a more rapid and sensitive response is unclear. One possibility is that the absence of the flexible N-terminal region (23–89) destabilizes the native rPrP conformation, allowing a more rapidly refolding into the amyloid conformation (Orrú et al., 2015a).

The introduction of the IQ-CSF protocol contributed to significant development of the assay by showing retained high specificity, increased sensitivity, and a reduced assay time compared to the previous QuIC (PQ) protocol (Table 3) (Orrú et al., 2015a,^2020^; Groveman et al., 2016; Park et al., 2016; Bongianni et al., 2017; Foutz et al., 2017; Franceschini et al., 2017; Rudge et al., 2018; Abu-Rumeileh et al., 2019; Fiorini et al., 2020; Rhoads et al., 2020; Vallabh et al., 2020; Xiao et al., 2020; Moško et al., 2021). In this regard, the IQ-CSF protocol showed a lower detection limit for seeding prions in a large patient cohort comprising the whole spectrum of human prions (Franceschini et al., 2017). Indeed, in this study, IQ-CSF correctly identified 81% of definite CJD cases tested negative by PQ-CSF, mainly including atypical and uncommon prion phenotypes such as VPSPr. Similarly, Rhoads and collaborators (Rhoads et al., 2020) obtained high sensitivity (90.3%) and specificity (98.5%) with IQ-CSF in a large study group, including 567 autopsy-verified cases (Table 3). Thanks to the large size of samples from patients with post-mortem histotyping or carrying *PRNP* mutations, the authors of the latter two studies identified several factors associated with the reduced sensitivity of RT-QuIC. Indeed, some atypical sporadic prion disease subtypes (i.e., VPSPr, sCJD VV1, MM2T, and MM2C), as well as some genetic prion diseases (i.e., FFI and GSS), are more likely to have false-negative RT-QuIC results (Franceschini et al., 2017; Rhoads et al., 2020).

**TABLE 3:** Performance of CSF-RT-QuIC in definite and/or probable sporadic prion disorders and controls (not-CJD and/or healthy subjects).

Substrate	n. cases	Sensitivity	n. Controls	Specificity	References
Hu rPrP23-231	59 sCJD	°83, 87, and 100%	179	100%	Atarashi et al., 2011
Ha PrP23-231	28 sCJD	79%	46	100%	Orrú et al., 2014
	11 sCJD	63%	1	100%	Orrú et al., 2015a
	109 sCJD	73%	64	100%	Groveman et al., 2016
	81 sCJD	77%	100	100%	Park et al., 2016
	276 sCJD 1 VPSPr	82% 0%	348	99%	Lattanzio et al., 2017
	50 sCJD	73%	–	–	Bongianni et al., 2017
	75 sCJD	89%	64	100%	Rudge et al., 2018
	65 sCJD	89%	118	100%	Hermann et al., 2018
	80 sCJD 1 VPSPr	83% 0%	109	100%	* Abu-Rumeileh et al., 2019
	65 sCJD	89%	20	NR	Zerr et al., 2020
Ha rPrP90-231	48 sCJD	96%	39	100%	Orrú et al., 2015a
	109 sCJD	95%	64	100%	Groveman et al., 2016
	22 sCJD	86%	17	100%	Bongianni et al., 2017
	189 sCJD 3 VPSPr	93% 100%	100	100%	Franceschini et al., 2017
	176 sCJD	93%	82	99%	Foutz et al., 2017
	62 sCJD 1 VPSPr	97% 100%	53	100%	* Abu-Rumeileh et al., 2019
	102 sCJD	96%	80	100%	* Fiorini et al., 2020
	444 sCJD 3 VPSPr	92% 67%	70	99%	* Rhoads et al., 2020
	30 sCJD	97%	30	100%	Xiao et al., 2020
	55 sCJD	96–100%	45	100%	Orrú et al., 2020
	26 s/gCJD^§^	88%	16	100%	Vallabh et al., 2020
Ha-Sh rPrP14-234	64 sCJD	80%	400	99%	Cramm et al., 2016
Bv rPrP23-231	26 s/gCJD^§^	53%	16	100%	Vallabh et al., 2020
	79 sCJD	89%	79	91%	^¥^ Mok et al., 2021

°Based on 3 cohorts. ^§^ The presentation of the results did not allow the distinction between CJD (n = 22) and gCJD (n = 4). *Indicate publications from the same group with partial overlap with previously published data. ^¥^Used post-mortem CSF samples.

*CJD, Creutzfeldt–Jakob disease; sCJD, sporadic CJD; gCJD, genetic CJD; rPrP, recombinant prion protein; Hu, human; Ha, hamster; Sh, sheep; Bv, bank vole; VPSPr, Variable Protease Sensitive Prionopathy; NR, not reported.*

Two additional substrates, the sheep-hamster chimeric rPrP and in particular the bank vole (Bv) rPrP23-230 (Cramm et al., 2015; Orrú et al., 2015b; Mok et al., 2021), demonstrated a diagnostic potential despite their high reactivity and self-aggregating predisposition. Bank voles and transgenic mice expressing Bv rPrP are highly susceptible to many prion strains (Nonno et al., 2006; Watts et al., 2014). Following a first successful attempt to convert BSE prions in the skin of 2 vCJD patients using Bv rPrP (Orrú et al., 2017), in a recent study, 1 out 2 CSF samples collected post-mortem from vCJD cases was also positively converted by Bv rPrP (Mok et al., 2021). However, the calculated sensitivity (88.6%) and specificity (91.2%) in the overall study cohort were below the mean of those reported in previous studies using traditional substrates (Table 3). The use of CSF samples obtained post-mortem represents a significant limitation of this study and makes the comparison with the results obtained with CSF collected in vitam difficult. Nevertheless, the results raise interest in using Bv rPrP substrate for prion strains that are not responsive to other conventional substrates, such as the BSE strain. It also emphasizes the need for further assay optimization and validation for routinely diagnostic uses to minimize false-positive results (Mok et al., 2021).

Finally, differences in the substrate production methods and refolding procedures may affect the performance of RT-QuIC (Cheng et al., 2018). In this study, the authors linked different rPrP production methods to their performance in RT-QuIC assay. They reported that Ha rPrP provided consistent results regardless of the investigated production procedures. The high tolerance of Ha rPrP to different production protocols may explain the robust and reproducible performance of RT-QuIC obtained in international studies in which various homemade Ha rPrP substrates were used (Cramm et al., 2016; McGuire et al., 2016; Orrú et al., 2020). However, given the effect that various rPrP production protocols have on RT-QuIC performance, caution is required when comparing inter-laboratory results.

### The Effect of Tissue Matrix: Urine, Plasma, Cerebrospinal Fluid, Olfactory Mucosa, Skin, and Eye Components

Currently, CSF is the matrix of choice for the prion RT-QuIC. The high performance of the CSF assay, the fact that CSF represents the accessible fluid that is more proximal to the brain, where PrPSc predominantly accumulates, and the systematic collection of this biofluid in patients with RPDs for diagnostic purposes all contributed to this scenario.

However, despite the highly dominant tropism of prions for the CNS, it is well-established that PrPSc may also accumulate in extra-neural tissues. Given the relatively low amount of PrPSc deposition in peripheral tissues, the detection of prions outside the CNS requires the use of ultrasensitive techniques, such as enhanced chemiluminescence (Head et al., 2003; Zanusso et al., 2003; Favereaux et al., 2004; Orrú et al., 2017; Baiardi et al., 2019b), tissue precipitation with sodium phosphotungstic acid (Glatzel et al., 2003; Zanusso et al., 2003), or other concentration/purification protocols (Head et al., 2003; Orrú et al., 2017; Baiardi et al., 2019b). Using these strategies, several groups detected PrPSc in the olfactory and retinal epithelium, cranial and peripheral nerves, muscles, skin, and spleen of sCJD patients. Moreover, they demonstrated PrPSc accumulations in many peripheral tissues, including tonsils, spleen, lymph nodes, circulating lymphocytes, and thymus in patients with vCJD (Wadsworth et al., 2001; Notari et al., 2010). These studies, performed in the pre-RT-QuIC era, paved the way for the application of the assay to accessible peripheral tissues collected in vitam. Indeed, several studies confirmed the ability of RT-QuIC to amplify a meager amount of PrPSc in peripheral tissue homogenates [i.e., olfactory mucosa (OM), and skin obtained by nasal brushing and punch biopsy, respectively] (Table 4). In sCJD, one group reported a sensitivity ranging from 90.5 to 96.5% and a specificity of 100% for the OM RT-QuIC using the Ha rPrP23-231 substrate (Orrú et al., 2014; Bongianni et al., 2017; Fiorini et al., 2020), suggesting an excellent diagnostic accuracy. Skin also represents a valuable matrix for the RT-QuIC assay (Orrú et al., 2017; Mammana et al., 2020). One study demonstrated that the PrPSc signal from both sCJD and vCJD skin samples taken *ex vivo* could be efficiently amplified by RT-QuIC using Bv rPrP as a substrate (100% sensitivity when multiple sites are analyzed). In contrast, only the PrPSc from sCJD skin samples reacted positively with the Ha rPrP23-231 substrate (Orrú et al., 2017). Moreover, the results of this study indicated that prion seeding activity was approximately 1000-fold lower in the skin than in the brain of the same patient and that the skin contains prion seed concentrations similar to those found in nasal brushings (Orrú et al., 2017). In another study, the skin RT-QuIC performed on punch biopsies taken both *ex vivo* and in vitam confirmed the high diagnostic value of the assay (Mammana et al., 2020) (Table 4). However, the Bv rPrP (88.6% sensitivity and 100% specificity) showed a better performance compared to Ha rPrP23-231 (68.6% sensitivity and 100% specificity). Moreover, although very preliminary, evidence also suggests that the prion-seeding activity in the skin increases with disease progression (Mammana et al., 2020). Recently, Xiao et al. (2020) applied the IQ protocol to skin samples collected in vitam from patients with probable sCJD and controls. They obtained a good sensitivity but a suboptimal specificity (Table 4).

**TABLE 4:** Performance of RT-QuIC in peripheral tissue matrices from definite and/or probable cases and controls (not-CJD and/or healthy).

Substrate	Matrix	n. cases	Sensitivity	n. controls	Specificity	References
Hu rPrP23-231	DT^#^	4 sCJD 1 gCJD 1 GSS^γ^	100%*	–	–	Satoh et al., 2019
Ha rPrP23-231	OM	29 sCJD 2 gCJD	97% 100%	43	100%	Orrú et al., 2014
	OM	61 sCJD 5 gCJD 2 GSS	90–95%^φ^ 75–80%^φ^ 50%	50	100%	Bongianni et al., 2017
	OM	35 sCJD	91%	7	100%	Fiorini et al., 2020
	Skin^§^	35 sCJD	69%	37	100%	Mammana et al., 2020
Ha rPrP90-231	Eyes^#^	11 sCJD	100%	6	100%	Orrú et al., 2018
	PNS^#^	12 sCJD	100%	2	100%	Baiardi et al., 2019b
	OM	9 sCJD	89–100%	19	95–100%	Orrú et al., 2020
	Skin	34 sCJD	91%	14	86%	Xiao et al., 2021
Bv rPrP90-231	OM	2 FFI	100%	26	100%	Redaelli et al., 2017
Bv rPrP23-231	Skin^#^	21 sCJD 2 vCJD	100%*	15	100%	Orrú et al., 2017
	Skin^§^	35 sCJD	89%	37	100%	Mammana et al., 2020

*When multiple sites are considered in the evaluation of the positive vs. negative outcome. Individual samples were occasionally negative. ^φ^ Depending on the type of brush used. ^γ^ P102L mutation. ^#^Collected post-mortem. ^§^ Collected both post-mortem and in vitam.

*CJD, Creutzfeldt–Jakob disease; sCJD, sporadic CJD; gCJD, genetic CJD; vCJD, variant CJD; FFI, fatal familial insomnia; GSS, Gerstmann–Sträussler–Scheinker disease; rPrP, recombinant prion protein; DT, digestive tract; OM, olfactory mucosa; PNS, peripheral nervous system; Hu, human; Ha, hamster; Bv, bank vole.*

A study demonstrated positive post-mortem RT-QuIC results on multiple eye components, including the retina, optic nerve, extraocular muscle, choroid, lens, vitreous, and sclera of 11 sCJD patients (Orrú et al., 2018). The assessment of the diagnostic value of the analysis of routinely accessible eye tissue or fluid needs further studies and validation. Besides cranial nerves, prion seeding activity has been demonstrated in peripheral nerves (sural, sciatic, femoral, and common peroneal) collected post-mortem from one gCJD-V210I and 11 sCJD individuals (Baiardi et al., 2019b). Both studies used Ha rPrP90-231 as recombinant substrate and reported complete sensitivity and specificity of the RT-QuIC assay (Orrú et al., 2018; Baiardi et al., 2019b). The observation that in scrapie-infected hamsters and transgenic mice, skin samples reveal prion seeding activity before the onset of overt clinical signs of infection (Wang et al., 2019) suggests that skin punch biopsies could be relevant for preclinical diagnosis of human prion disease, particularly in subjects at genetic risk for the disease.

Besides the skin punches and the brushing of OM, digestive tract biopsies may provide an accessible tissue source for the detection of prion seeding activity. Satoh et al. (2019) recently demonstrated a 100% sensitivity in a few patients with sCJD (*n* = 4) and genetic prion disease (*n* = 2) using post-mortem tissue and multiple sites for each patient (Table 4). The RT-QuIC assay is far less efficient than other cell-free *in vitro* seeding assays, such as protein misfolding cyclic amplification and its adaptations, in detecting vCJD prions (Giaccone and Moda, 2020). Orrú et al. (2011) tried to improve the assay by adding an initial immunoprecipitation step to the RT-QuIC protocol and replenishing the rPrP (chimeric sheep-hamster) at 24 h. The amended assay, called enhanced QuIC, allowed PrPSc detection in human plasma samples spiked with vCJD brain homogenates up to 10^14^-fold dilutions, and showed significantly more sensitivity than the standard protocol (Orrú et al., 2011). However, the higher technical complexity of enhanced QuIC, compared with protein misfolding cyclic amplification, prevented its reproducibility, standardization, and diffusion.

### Real-Time Quaking-Induced Conversion and the Diagnostic Criteria for Sporadic Creutzfeldt–Jakob Disease

Shortly after its application to clinical cohorts, the valuable performance of RT-QuIC in discriminating sCJD from non-CJD with exceptionally high specificity prompted the update of the diagnostic criteria for sCJD. Since January 2017, the positivity of RT-QuIC assay in CSF or other tissues in subjects presenting with a progressive neurological syndrome supports the clinical diagnosis of probable sCJD (Table 2), making no longer necessary the previous stringent clinical criteria for “possible” CJD (i.e., the association between dementia and multifocal neurological signs) as mandatory for accepting the diagnosis of probable disease based on the positivity of electroencephalography, CSF or MRI biomarkers. Indeed, the latter clinical scenario is uncommon at onset and during the early disease stage, especially in the “atypical” sCJD subtypes (all but the MM(V)1), which account for up to 40% of cases. Therefore, the association between positive RT-QuIC assay and a non-further specified “progressive neurological syndrome” allows to diagnose the disease earlier and more accurately even in cases presenting “unusual” neurological symptoms and/signs or isolated symptoms such as in the Heidenhain and the ataxic (VV2) variants (Baiardi et al., 2018).

Nevertheless, despite the addition of RT-QuIC, the novel criteria still include the diagnostic investigations of the previous criteria (Zerr et al., 2009), given their wider diffusion (i.e., at least DW-MRI, and electroencephalography are often available in secondary care centers). They presumably have an added diagnostic value in cases tested negative by RT-QuIC. However, to some extent, they also increase the false positive cases, given their incomplete specificity. Future studies should carefully address this issue and evaluate if the insufficient specificity of the other biomarkers exceeds the limit in sensitivity of the RT-QuIC.

The variable protocols used among laboratories, including the different recombinant substrates known to affect the test performance, represent another issue to address. Notably, current updated diagnostic criteria do not include any technical specification or detail in this regard.

Finally, the choice to keep the door open to matrices other than CSF for diagnostic RT-QuIC deserves a further comment. To date, CSF is by far the most studied matrix. CSF RT-QuIC using different recombinant substrates showed reproducible results among research groups and high inter-laboratory agreement in ring trials (McGuire et al., 2016; Orrú et al., 2020). Conversely, despite the promising initial results, RT-QuIC using alternative matrices, such as OM and skin, is still limited to a few specialized laboratories, confirmatory studies are lacking, and the performance of different recombinant substrates are largely unknown. For these reasons, RT-QuIC assays currently use CSF for diagnostic purposes, even if other specimens are allowed according to the official criteria (Hermann et al., 2021).

## Effect of the Disease Subtype on Real-Time Quaking-Induced Conversion Performance

### Sporadic Prion Disease

The vast phenotypic heterogeneity of human prion diseases has profound implications for the clinical diagnosis, including the results of ancillary investigations, which consistently show variable accuracy depending on the disease phenotype. Here we reviewed the results of studies applying the RT-QuIC on CSF or peripheral tissue samples collected in vitam or post-mortem in neuropathologically confirmed sCJD cases classified according to the disease histotype (Table 5). Since the description of mixed histotypes is scanty in most studies, we considered only those clearly stating the dominant histotype of mixed cases. Similarly, we only included MM2 subjects when we found a clear statement on the distinction between the cortical and thalamic subtypes.

**TABLE 5:** Diagnostic performance of the RT-QuIC assay across the spectrum of sCJD subtypes.

		MM(V)1		VV2		MV2(K)		MM2C		MM2T		VV1	
References	Prot.	Pos/T	%	Pos/T	%	Pos/T	%	Pos/T	%	Pos/T	%	Pos/T	%
**CSF**													
Orrú et al., 2014	PQ	9/11	82	1/1	100	1/1	100	–	–	–	–	–	–
Bongianni et al., 2017	PQ	14/15	93	–	–	1/2	50	–	–	–	–	–	–
Lattanzio et al., 2017	PQ	95/107	89	21/27	78	21/26	81	4/9	44	1/3	33	1/1	100
Hamaguchi et al., 2020	PQ	16/17	94	–	–	–	–	2/5	40	1/4	25	–	–
McGuire et al., 2016 **^§^**	PQ	2/2	100	–	–	–	–	–	–	–	–	–	–
Total		136/152	90	22/28	79	23/29	79	6/14	43	2/7	29	1/1	100

Bongianni et al., 2017	IQ	1/1	100	–	–	0/2	0	–	–	–	–	–	–
Franceschini et al., 2017	IQ	40/43	93	33/33	100	24/26	92	6/9	66.7	3/4	75.0	1/1	100
Foutz et al., 2017	IQ	100/105	95	26/26	100	8/9	89	7/9	78	0/2	0	6/8	75
Rhoads et al., 2020	IQ	195/205	95	54/56	96	37/40	92	18/23	78	0/5	0	0/3	0
Orrú et al., 2020 **^§^**	IQ	24/24	100	2/2	100	4/4	100	–	–	–	–	–	–
Total		360/378	95	115/117	98	73/81	90	31/41	76	3/11	27	7/12	58

**Skin**													
Orrú et al., 2017	Bv	7/7	100	4/4	100	1/1	100	–	–	–	–	1/1	100
Mammana et al., 2020	Bv	17/20	85	3/3	100	2/2	100	2/3	66.7	–	–	–	–
Total		24/27	89	7/7	100	3/3	100	2/3	66.7	–	–	1/1	100

Mammana et al., 2020	PQ	8/20	40.0	3/3	100	2/2	100	2/3	66.7	–	–	–	–
**Olfactory mucosa**													
Orrú et al., 2014	PQ	11/11	100	1/1	100	1/1	100	–	–	–	–	–	–
Bongianni et al., 2017	PQ	16/16	100	–	–	3/3	100	–	–	–	–	–	–
Total		27/27	100	1/1	100	4/4	100						

Orrú et al., 2020 **^§^**	IQ	2/2	100	1/1	100	–	–	–	–	–	–	–	–
**Nerves**													
Baiardi et al., 2019b	IQ	4/4	100	5/5	100	1/1	100	1/1	100	–	–	–	–

*^§^ Ring trial.*

*Prot., protocol; Pos/T, positive/tested; PQ, first generation “previous” QuIC (Ha rPrP 23-231); IQ, second generation “improved” QuIC (Ha rPrP 90-231); Bv, bank vole.*

#### Typical Forms (MM1/MV1 Histotype)

Overall, the RT-QuIC assay showed excellent sensitivity in detecting prion seeding activity in this histotype, with only minor variations determined by the rPrP substrate and the matrix analyzed (Table 5). Using CSF, the PQ protocol yielded a slightly lower sensitivity (88.8–94.1%) (Bongianni et al., 2017; Lattanzio et al., 2017; Hamaguchi et al., 2020) compared to IQ-CSF (93.0–100%) (Bongianni et al., 2017; Foutz et al., 2017; Franceschini et al., 2017; Rhoads et al., 2020). Moreover, two independent ring trials assessing the agreement among laboratories demonstrated a complete concordance of RT-QuIC results and 100% sensitivity for both these recombinant substrates (McGuire et al., 2016; Orrú et al., 2020).

Two studies from the same group analyzed the OM obtained with nasal brushing in 11 and 16 patients with sCJD MM(V)1 and reported 100% assay sensitivity (Ha rPrP23-231) (Orrú et al., 2014; Bongianni et al., 2017). The results of a ring trial including six laboratories that tested the OM of two MM1 patients using Ha rPrP90-231 showed a complete concordance among laboratories (Orrú et al., 2020). Two research groups tested RT-QuIC in skin samples from MM(V)1 taken post-mortem (Orrú et al., 2017; Mammana et al., 2020) using the Bv rPrP as substrate. The two studies reported a discrepant sensitivity (7/7, 100% vs. 17/20, 85.0%) likely related to the different anatomic location (lower back/apex/area near the ear in one study vs. cervical/thigh in the other) and/or the number of skin samples analyzed for each patient. Indeed, no single skin location was always RT-QuIC-positive, even in the study reporting a positivity in all patients. Pursuing the excellent results obtained on skin biopsies collected post-mortem, one study also exploited the RT-QuIC value with skin samples taken in vitam (Mammana et al., 2020). A single MM1 patient underwent the punch biopsy both in vitam and *ex vivo*, and gave a positive result in both skin samples collected at cervical C7 level (Mammana et al., 2020). Additionally, Mammana et al. (2020) compared the Ha rPrP23-231 and Bv rPrP substrates, demonstrating a better performance of the latter in the MM(V)1 histotype (sensitivity 40.0% vs. 85.0%). In a single study, all peripheral nerves (sural *n* = 1, sciatic *n* = 3) samples taken post-mortem from MM(V)1 subjects tested by the IQ protocol revealed a positive prion seeding activity (Baiardi et al., 2019b).

#### Ataxic Forms (VV2 and MV2K Histotypes)

There was a strong agreement between three studies reporting a high sensitivity of the IQ-CSF for the VV2 subtype (96.4–100%), which was only slightly lower for MV2K individuals (88.9–92.5%) (Table 5) (Foutz et al., 2017; Franceschini et al., 2017; Rhoads et al., 2020). A recent ring trial confirmed the result on VV2 cases (Orrú et al., 2020). In contrast, the only study testing these histotypes with PQ-CSF in a significant number of patients reported a suboptimal performance in both groups (sensitivity 77.8% in VV2 and 80.8% in MV2K) (Lattanzio et al., 2017). The few cases tested (VV2, *n* = 7; MV2K, *n* = 3) in the skin (Orrú et al., 2017; Mammana et al., 2020) by two independent groups using Bv rPrP reported a positive outcome in all VV2, and MV2K samples collected post-mortem, irrespectively of their anatomic location (Orrú et al., 2017; Mammana et al., 2020). In contrast, the single MV2K patient examined *in vivo* showed prion seeding activity only in the cervical area (Mammana et al., 2020). Of note, BvPrP and Ha rPrP23-231 showed an identical diagnostic accuracy in these histotypes (Mammana et al., 2020).

Information about OM RT-QuIC in ataxic sCJD subtypes is limited. OM from a single VV2 patient has been included in the already mentioned IQ ring trial and tested positive by all participants (Orrú et al., 2020). A single OM was also positive with the PQ protocol (Orrú et al., 2014).

#### Cortical Forms (MM2C and VV1 Histotypes)

Given their rarity, a limited number of MM2C and VV1 cases underwent RT-QuIC testing. Both PQ- and IQ-CSF showed a suboptimal sensitivity in these histotypes. The substrate also influenced the results. In particular, about 70% of MM2C tested positive by IQ-CSF (range 66.7–78.3%) (Foutz et al., 2017; Franceschini et al., 2017; Rhoads et al., 2020) compared to about 40% of those analyzed by PQ-CSF (range 40.0–44.4%) (Lattanzio et al., 2017; Hamaguchi et al., 2020) (Table 5). VV1 individuals displayed an even more significant variability across studies (Foutz et al., 2017; Franceschini et al., 2017; Rhoads et al., 2020). Overall, 7 out 12 cases (58.3%) tested by IQ-CSF have been reported positive. Interestingly, as for PQ in the brain (McGuire et al., 2012; Peden et al., 2012), Franceschini et al. (2017) documented a prolonged lag phase and lower fluorescence peak in MM2C by using IQ-CSF. These findings indirectly suggest that RT-QuIC reactivity is lower in this histotype than in MM(V)1, VV2, and MV2K, independently from the recombinant substrate. Two out of 3 skin samples from MM2C patients analyzed by Mammana et al. (2020) gave positive responses, irrespectively of the anatomic site and recombinant substrate (Bv or Ha rPrP23-231). Orrú et al. (2017) reported a positive RT-QuIC reaction in the only VV1 skin sample they tested. The sural nerve of an MM2C patient also showed prion seeding activity using the IQ protocol (Baiardi et al., 2019b).

#### Sporadic Fatal Insomnia (MM2T Histotype)

Data are available only for a few cases, given the low prevalence of this histotype. As for FFI, RT-QuIC showed an overall low sensitivity in sCJD MM2T. Two out of 7 (28.6%) (Lattanzio et al., 2017; Hamaguchi et al., 2020) cases tested positive by PQ-CSF, and in 3 of 11 (27.3%) by IQ-CSF (Foutz et al., 2017; Franceschini et al., 2017; Rhoads et al., 2020) (Table 5).

### Genetic Prion Diseases

The anamnestic positive family history or the presence of a pathogenic *PRNP* mutation in a patient with a progressive neuropsychiatric syndrome already supports the clinical diagnosis of genetic prion disease. However, this diagnosis can be complex sometimes due to the wide range of clinicopathological presentations, the very low incidence, and the possibility of a negative familial history even for mutations with high penetrance (Ladogana and Kovacs, 2018). In this context, the novel ultrasensitive seeding assays contributed to a more accurate recognition of patients with genetic prion disease. As for the sporadic form, the diagnostic value of RT-QuIC differs according to the mutation and clinicopathological phenotype. Overall, the diagnostic accuracy is high in gCJD and low in GSS and FFI cases. Finally, RT-QuIC might be of pivotal importance to determine when an eventual preventive therapy should be started in future preventive clinical trials in people at risk for genetic prion diseases (Hermann et al., 2020).

#### Genetic Creutzfeldt–Jakob Disease

In gCJD associated with E200K-129M and V210I-129M, the CSF prion RT-QuIC showed a sensitivity ranging from 81.8 to 100% (i.e., in studies with *n* ≥ 5), depending on the protocol applied and the *PRNP* haplotype (Sano et al., 2013; Cramm et al., 2015; Foutz et al., 2017; Franceschini et al., 2017; Lattanzio et al., 2017) (Table 6).

**TABLE 6:** Performance of CSF-RT-QuIC in genetic prion diseases according to phenotype and *PRNP* mutation.

Recombinant substrate	n. cases	Sensitivity	n. cases – *PRNP* mutation*	Sensitivity	References
Hu rPrP23-231	24 gCJD 12 FFI 20 GSS	83% 83% 90%	22 E200K, 2 V203I 20 P102L	82%, 100% 90%	Sano et al., 2013
Ha rPrP23-231	2 gCJD	50%	2 E200K	50%	Orrú et al., 2014
	3 gCJD 1 GSS	67% 0%	2 V210I, 1 E200K 1 P102L	50%, 100% 0%	Groveman et al., 2016
	46 gCJD	91%	21 V210I, 20 E200K, 2 D178N, 1 R208H, 1 4-OPRI, 1 V203I	95%, 100%, 0% 0%, 100%, 100%	Lattanzio et al., 2017
	6 gCJD 1 GSS	67% 0%	3 V210I, 2 E200K, 1 V180I 1 P102L	67%, 100%, 0% 0%	Bongianni et al., 2017
Ha rPrP90-231	3 gCJD 1 GSS	67% 0%	2 V210I, 1 E200K 1 P102L	50%, 100% 0%	Groveman et al., 2016
	2 gCJD	50%	1 V210, 1 V180I	100%, 0%	Bongianni et al., 2017
	33 gCJD 2 FFI 6 GSS	100% 0% 33%	20 E200K, 10 V210I, 1 D178N, 1 R208H, 1 E219G 3 P102L, 1 A117V, 1 D202N, 1 8-OPRI	100%, 100%, 0% 0%, 0% 33%, 100%, 0%, 0%	Franceschini et al., 2017
	14 gCJD 1 FFI 2 GSS	100% 100% 50%	12 E200K, 2 V210I 1 P102L, 1 A117V	100%, 100% 100%, 0%	Foutz et al., 2017
	31 gCJD 3 GSS	97% 33%	21 E200K, 7 V210I, 1 A133V, 1 V203I, 1 2-OPRI 2 P102L, 1 A117V	n.s. n.s.	Rhoads et al., 2020
Bv rPrP23-231	13 gCJD 7 GSS	54% 14%	6 E200K, 4 6-OPRI, 2 4-OPRI, 1 E196K 5 P102L, 2 A117V	50%, 75%, 0%, 100% 20%, 0%	^¥^ Mok et al., 2021
Ha-Sh rPrP14-234	39 gCJD 7 FFI	100% 57%	33 E200K, 6 V210I	100%, 100%	Cramm et al., 2016

*PRNP mutation in the FFI group is omitted because of the disease is uniquely associated with the D178N-129M haplotype. When reported in the gCJD group the D178N mutation is invariably in cis with 129V. ^¥^Used CSF samples taken post-mortem. Mok et al. (2021) analyzed two case carrying the D178N mutation, but neither the haplotype nor the clinical phenotype was specified; therefore, we omitted them from the table.

*gCJD, genetic Creutzfeldt–Jakob disease; FFI, fatal familial insomnia; GSS, Gerstmann–Sträussler–Scheinker disease; Hu, human; Ha, hamster; Sh, sheep; n.s. not specified.*

Among subjects carrying rare mutations, the CSF from a patient with the V180I substitution, and two with the D178N-129V haplotype gave a negative response. In contrast, the CSF from patients carrying V203I, E219G and 4-OPRI were positive (Sano et al., 2013; Franceschini et al., 2017; Lattanzio et al., 2017). As in sCJD, overall, the IQ-CSF showed a slightly higher sensitivity than the PQ-CSF protocol.

In two studies, patients carrying the E200K-129M haplotype showed a significantly shorter lag phase than those carrying other mutations in *cis* with 129M and those with sCJD MM(V)1 (Cramm et al., 2015; Franceschini et al., 2017).

In line with the studies mentioned above, we found a positive PrPSc seeding activity with the PQ protocol in 93.4% of the 106 tested individuals collected at the Istituto Superiore di Sanità, including both prevalent and rare point mutations (Table 7). The assay sensitivity was slightly higher in patients carrying the E200K-129M (96.0%) haplotype than those with V210I-129M (94.2%).

**TABLE 7:** CSF RT-QuIC sensitivity in the ISS cohort of genetic prion diseases.

Phenotype	*PRNP* mutation	n. cases	n. of positive	Sensitivity
Genetic CJD	V210I	69	65	94%
	E200K	25	24	96%
	R208H	8	8	100%
	V203I	2	1	50%
	E196K	1	1	100%
	V180I	1	0	0%
GSS	P102L	3	1	33%
Atypical	7-OPRI	1	0	0%

*ISS, Istituto Superiore di Sanità, Italy.*

#### Gerstmann–Sträussler–Scheinker Disease

Gerstmann–Sträussler–Scheinker disease is associated with several point or insert mutations (Ghetti et al., 2018). Numerous studies demonstrated that CSF surrogate biomarkers are less useful in the GSS diagnostic workup than in gCJD. The same low sensitivity extends to the prion RT-QuIC assay (Green and Zanusso, 2018), although the relatively low number of cases analyzed due to the low prevalence of disease does not allow a definite conclusion on this issue. The RT-QuIC assay applied to CSF samples from patients carrying the most common GSS-associated mutation (P102L) showed a highly variable positivity. Intriguingly, the PQ-CSF (Hu rPrP23-231) performed better (18/20 positive samples, 90%) (Sano et al., 2013) than the IQ-CSF assay or using Bv rPrP. However, the well-known phenotypic heterogeneity of patients carrying the P102L mutation, comprising two phenotypes with distinct molecular pathology and disease progression (Parchi et al., 1998), makes it difficult to compare the results between GSS cohorts lacking a detailed phenotypic characterization. Indeed, it is expected that, between the two phenotypes, the one with CJD-like features (i.e., with PrPSc type 1 deposition and spongiform change) would be associated with higher prion seeding activity in both brain and CSF.

Testing OM samples yielded a 50% positivity in two GSS-P102L (Bongianni et al., 2017). Regarding the other GSS-linked mutations, the IQ-CSF was negative in a single patient carrying the D202N mutation, in a patient carrying an 8-OPRI, and in three out four cases carrying the A117V mutation (Foutz et al., 2017; Franceschini et al., 2017; Mok et al., 2021).

#### Fatal Familia Insomnia

A single study with CSF samples from patients with FFI successfully showed seeding activity in RT-QuIC reactions using Hu rPrP23-231 with 83.3% positivity (Sano et al., 2013). This level of sensitivity was not replicated in reactions seeded by CSF using chimeric sheep-hamster rPrP in another study (Cramm et al., 2016). The IQ-CSF also gave negative results in two FFI cases (Franceschini et al., 2017). In contrast, prion seeding activity has been detected in vitam in the OM harvested from two FFI patients using truncated Bv rPrP90-231 as substrate (Redaelli et al., 2017).

### Acquired Prion Diseases

#### Iatrogenic Creutzfeldt–Jakob Disease

Only a few studies are available regarding the use of RT-QuIC assays to diagnose iCJD. In 22 cases of iCJD secondary to human cadaveric growth hormone administration, RT-QuIC assay showed CSF seeding activity in 14 patients, yielding a sensitivity of 63.6% using PQ-CSF (Green and Zanusso, 2018). In a second more recent study CSF RT-QuIC was positive in 16/18 dura mater graft cases, 1/2 human cadaveric growth hormone cases, and 1 case of corneal transplant (overall sensitivity 85.7%) (Llorens et al., 2020).

#### Variant Creutzfeldt–Jakob Disease

Pathological PrP from vCJD brain and CSF did not generate seeding activity using Ha rPrP23-231. The enhanced RT-QuIC assay with Bv rPrP supported PrPSc amplification from vCJD brain tissue but not CSF. A recent study reported positivity in 1/2 vCJD cases in post-mortem CSF (Mok et al., 2021). Two out of two skin samples tested positive using Bv rPrP (Orrú et al., 2017).

## Specificity, Protocol Heterogeneity, and Interlaboratory Reproducibility of the Prion Real-Time Quaking-Induced Conversion

Prion RT-QuIC indirectly detects the presence of PrPSc; therefore, the positive seeding activity revealed by the test represents a putative disease-specific marker. However, the requirement of an excess of rPrP in a mixture favoring the seeded aggregation of the protein poses a potential risk for a spontaneous substrate aggregation generating false-positive signals. Consequently, samples are loaded at least three times (usually four) and are called positive when the signal of at least two replicates exceeds a pre-set threshold. Moreover, negative control samples are loaded in each plate.

All studies comprising many control samples have reported an assay specificity close to 100%. All but one of the five CSF “control” (i.e., with a clinical diagnosis of non-CJD) samples tested positive by prion RT-QuIC only had a clinical diagnosis (Vascular dementia, Frontotemporal dementia, paraneoplastic syndrome, and Alzheimer’s disease) and were lost at follow up (McGuire et al., 2012; Cramm et al., 2016; Lattanzio et al., 2017). The only post-mortem diagnosis was a neuropathologically definite Dementia with Lewy bodies. A low amount of PrPSc was detected by Western blotting and conformational-dependent immunoassay (Foutz et al., 2017), raising the possibility that the patient had a subclinical prion disease in addition to Dementia with Lewy bodies. In conclusion, although false readouts by prion RT-QuIC seem to be extremely rare, and some of them may even represent clinically unrecognized prion cases, the test does not seem to provide yet a level of diagnostic certainty comparable to a neuropathological examination. Consequently, currently updated diagnostic criteria still require a post-mortem examination for the definite diagnosis of sporadic or acquired prion disease (Hermann et al., 2021). It will be essential to monitor the occurrence of false-positive results in neuropathologically verified samples and all potential causing factors.

On a different critical issue, the assay includes several analytical steps that can affect its reproducibility among laboratories. Above all, differences in the substrate production methods and refolding procedures may influence the performance of the RT-QuIC test (Cheng et al., 2018). Nevertheless, the results of international studies showing a high concordance between laboratories seem to indicate a limited influence of such variables on the diagnostic performance of the assay (Cramm et al., 2016; McGuire et al., 2016).

Despite the use of different rPrP substrates [full-length hamster rPrP (Bristol, United Kingdom), home-made human rPrP, sheep-hamster chimeric hamster], there was optimal agreement among seven (first ring trial) and eleven (second ring trial) participating laboratories in one study (McGuire et al., 2016). Similarly, in the second study of this kind, the multi-center inter-laboratory reproducibility of RT-QuIC revealed a Fleiss’ kappa value of 0.83 (95% CI: 0.4–1.00), indicating an almost perfect agreement (Cramm et al., 2016). An aid to overcome the issue of the reproducibility of the PQ-CSF and to facilitate the acquisition of the technical skills to perform it in an increasing number of laboratories may be provided by a technical report issued in February 2021 by the European Centre for Disease Prevention and Control on the standard operating procedures of the assay from the recombinant production to the CSF analysis (European Centre for Disease Prevention and Control [ECDC], 2021). Finally, a recent study also tested the interlaboratory reproducibility of the IQ-CSF. The ring trial, conducted between six laboratories examining CSF and OM samples, showed 98–100% concordance (Orrú et al., 2020).

## Conclusion and Future Challenges and Developments

After its original implementation 10 years ago, the RT-QuIC has become the most potent laboratory aid for the *in vivo* diagnosis of prion disease and has significantly impacted both clinical diagnostic criteria and clinical practice. Despite these significant advances, there is still room for further improvement of the assay and an expansion of its potential applications in clinical practice. Important limitations of the currently available prion RT-QuIC protocols are the incapacity to reliably distinguish between different prion strains and the reduced sensitivity toward disease forms associated with reduced transmissibility and prolonged disease duration. Indeed, as stated above, the assay has optimal sensitivity only for the two most common CJD strains (i.e., M1 and V2), which also are among the most readily transmissible, as shown by results of animal and interhuman (i.e., iCJD) transmissions. Moreover, despite some initial claims (Foutz et al., 2017), current RT-QuIC set-ups are still associated with significant plate-to-plate variability, limiting the possibility of distinguishing between CJD subtypes and disease stages based on quantification of the kinetic parameters of the reaction. Future work should address these limitations. Unfortunately, the extreme rarity of most prion disease variants associated with low transmissibility is *per se* a limitation to the development of such studies. Nonetheless, attempts should be carried out to enhance further the reactivity between PrPSc and the substrate by testing novel recombinant protein sequences and reaction conditions. The prion RT-QuIC should also be tested as a prodromal biomarker for patients carrying a *PRNP* mutation linked to gCJD. Such prodromal biomarkers, which currently only exist for slowly progressive neurodegenerative disorders, will be of critical value in the future to offer opportunities for secondary prevention, enabling selective enrollment of individuals close to the clinical onset or allowing subclinical markers of disease to be tracked as outcome measures. For the latter use, efforts should also be made to improve the reliability of the RT-QuIC as a quantitative assay to measure a reduction in seeding activity correlating with other known prognostic variables or measuring a therapeutic response.

## Author Contributions

PP conceptualized the review and finalized the manuscript. All authors performed literature survey and drafted the manuscript. AP, SB, and AL prepared the tables. SB prepared the figure. All authors approved the submitted version.

## Conflict of Interest

The authors declare that the research was conducted in the absence of any commercial or financial relationships that could be construed as a potential conflict of interest.

## Publisher’s Note

All claims expressed in this article are solely those of the authors and do not necessarily represent those of their affiliated organizations, or those of the publisher, the editors and the reviewers. Any product that may be evaluated in this article, or claim that may be made by its manufacturer, is not guaranteed or endorsed by the publisher.

## Footnotes

1
https://www.eurocjd.ed.ac.uk/node/833

## Abbreviations

Bv: bank vole
BSE: bovine spongiform encephalopathy
CNS: central nervous system
CSF: cerebrospinal fluid
CJD: Creutzfeldt–Jakob disease
DW-MRI: diffusion-weighted magnetic resonance imaging
FFI: fatal familial insomnia
gCJD: genetic CJD
GSS: Gerstman–Sträussler–Scheinker disease
Ha: hamster
iCJD: iatrogenic
M: methionine
OPRI: octapeptide repeat insertion
OM: olfactory mucosa
PrP: prion protein
PrP*^Sc^*: PrP scrapie
PrP*^C^*: PrP cellular
RPDs: rapidly progressive dementias
RT-QuIC: real-time quaking-induced conversion
rPrP: recombinant PrP
sCJD: sporadic
ThT: thioflavin-T
V: valine
VPSPr: variably protease-sensitive prionopathy
vCJD: variant CJD.
